# The Diagnosis and Treatment of Gonorrhœal Arthritis

**Published:** 1907-06-15

**Authors:** J. Porter Parkinson

**Affiliations:** 57 Wimpole Street, W.


### EDITOR'S LETTER-BOX.

[Our Correspondents are reminded that prolixity is a great bar to publication, and that brevity of style and conciseness of statement greatly facilitate early insertion.]

### THE DIAGNOSIS AND TREATMENT OF GONORRHŒAL ARTHRITIS.

Sir,—In an article in this week's number of your journal, the treatment of the above-mentioned disease is alluded to, and a few lines are devoted to an entirely new method of treating it, first described by Dr. Fenwick and myself. In the description it seems to me that the two most essential points are not mentioned. These are, in brief: (1) The serum is given by rectal injection; and (2) It is with only one kind of polyvalent serum that we have obtained successful results—namely, that prepared in the Wellcome Laboratories, other varieties not being so successful owing, no doubt, to the strains of cocci being different. I think that if the treatment be mentioned, these essential facts should be given, otherwise the treatment may be considered to be useless, whereas Dr. Fenwick and I have proved its value over and over again. Hoping you will find space for this short explanation,

Believe me, yours truly,

J. Porter Parkinson.

57 Wimpole Street, W., June 8.

